# Neural components of altruistic punishment

**DOI:** 10.3389/fnins.2015.00026

**Published:** 2015-02-09

**Authors:** Emily Du, Steve W. C. Chang

**Affiliations:** ^1^Center for Cognitive Neuroscience, Duke Institute for Brain Sciences, Duke UniversityDurham, NC, USA; ^2^Department of Psychology, Yale UniversityNew Haven, CT, USA; ^3^Department of Neurobiology, Yale University School of MedicineNew Haven, CT, USA

**Keywords:** altruistic punishment, costly punishment, inequity aversion, cost-benefit calculation, social reference frame

## Abstract

Altruistic punishment, which occurs when an individual incurs a cost to punish in response to unfairness or a norm violation, may play a role in perpetuating cooperation. The neural correlates underlying costly punishment have only recently begun to be explored. Here we review the current state of research on the neural basis of altruism from the perspectives of costly punishment, emphasizing the importance of characterizing elementary neural processes underlying a decision to punish. In particular, we emphasize three cognitive processes that contribute to the decision to altruistically punish in most scenarios: inequity aversion, cost-benefit calculation, and social reference frame to distinguish self from others. Overall, we argue for the importance of understanding the neural correlates of altruistic punishment with respect to the core computations necessary to achieve a decision to punish.

## Punishment in a cooperative society

Hammurabi's Code is regarded as one of the world's oldest written legal systems, a timeless example from the ancient world of explicit, codified social norms and punishments instated against those who defected (Jarus, 2014). While most modern proceedings no longer follow *lex talionis*, today's societies continue to rely on cooperation between individuals to promote collective action, produce public goods, and deter free-riding (Ostrom, 2000).

Altruistic punishment occurs when an individual forgoes a personal gain to punish (Seymour et al., 2007). The biological definition of “altruism,” unlike its colloquial counterpart, does not assume or impose intentionality to the actors of altruistic behavior (Wilson, 1992). Here, “altruistic” describes the sacrifice of personal gain, not the motivation of this sacrifice. In second party (SP) punishment, an individual who receives an unfair offer in a monetary exchange or suffers from a selfish investment made by a partner punishes by reducing the norm violator's payout at a cost (Fehr and Gächter, 2002; Egas and Riedl, 2008), or rejects the unfair offer such that both players have a reduced payout (Güth and Tietz, 1990). Third party (TP) punishment, on the other hand, occurs when an uninvolved individual punishes the violator at a cost (Fehr and Fischbacher, 2004; Bernhard et al., 2006).

Altruistic punishment generates a vast array of questions about its behavioral and neural mechanisms. The evolutionary consequences for promoting prosocial behaviors, such as cooperation, remain continuously debated (Nakamaru and Iwasa, 2006; Rand et al., 2010; Rand and Nowak, 2013; Peysakhovich et al., 2014) but lie outside the scope of this paper; we instead focus on neural mechanisms that could be at play when deciding to costly punish. Our knowledge could benefit from a framework that incorporates neural computations contributing to altruistic punishment. Here, we explore the current literature on neuroscientific studies of operationally-defined altruistic punishment, and emphasize three cognitive processes that guide the decision to altruistically punish, namely inequity aversion, cost-benefit calculation, and social reference frame used for distinguishing self from others. These three cognitive processes have distinct neural correlates, as evidenced by literature that will be discussed, which could relate to the neural mechanism that underlies the complex decision of altruistic punishment. Therefore, connecting the literature on altruistic punishment to that of these three cognitive processes could inform our understanding of the neural correlates of punishment.

## Neural correlates of altruistic punishment

How does the brain mediate altruistic punishment? Many studies have directly explored this question (Table 1). Blood-oxygen-level dependent (BOLD) signals in the bilateral anterior insula (AI), dorsolateral prefrontal cortex (DLPFC), and anterior cingulate cortex (ACC) are all associated with receiving unfair vs. fair offers from another individual (Sanfey et al., 2003). Overall, converging evidence seems to suggest an involvement of reinforcement via the striatum in mediating altruistic punishment. One study compared BOLD activity of participants punishing as the SP and TP during a dictator game, and found differential activations in the right nucleus accumbens (NAc) and DLPFC (Strobel et al., 2011). Another study found greater activations in DLPFC and caudate nucleus (CdN) among other regions when individuals received unfair offers (Spitzer et al., 2007). Greater CdN activation has also been associated with costly compared to symbolic punishment (i.e., no reduction in endowment for either player), in which individuals' willingness to incur a greater cost to punish was associated with stronger CdN activations (de Quervain et al., 2004; White et al., 2014). Intriguingly, reducing serotonin signaling during the ultimatum game (UG) can lead to an increase in the likelihood of punishing others who responded to them unfairly by modulating striatal activations (Crockett et al., 2013), suggesting that serotonin may set the sensitivity threshold for fairness- and punishment-related processing.

**Table 1:** **Representative summary of neural basis of altruistic punishment and the involvement of neural processes underlying inequity aversion, cost-benefit calculation, and social reference frame**.

**Altruistic punishment**									
**References^a^**	**Phenomenon**	**Task**	**Conditions^b^**	**Technique**	**Main results**	**BOLD signal predictability**	**Inequity aversion**	**Cost-benefit calculation**	**Social reference frame**
Sanfey et al., 2003	Fairness	UG	U, F, NS	fMRI	U - F: AI, DLPFC, ACC	AI activation positively correlated with rejection of unfair offers	Reject unfair offers	Reject or accept offers	Personal payout vs. partner's payout
Strobel et al., 2011	Punishment, reward	DG	C, N, SP, TP	fMRI (predefined ROIs)	C - N: Cingulate gyrus, DLPFC, insula, CdN, NAc	Left DLPFC (SP) weaker activation in punished vs. not punished trials, and opposite in TP condition	Receive unfair offer	Pay to reduce partner's payout	Punish as second party or third party
					SP - TP: NAc, cingulate gyrus				
Spitzer et al., 2007	Punishment, social norm compliance	DG	C, N, NS	fMRI	C - N: CdN, DLPFC, VLPFC, OLPFC	OLPFC activation (C - NS) positively correlated with amount of monetary transfer and Machiavelli score. AI activation (C - NS) positively correlated with Machiavelli score	Receive unfair offers	Pay to reduce partner's payout or take no action	Personal payout vs. partner's payout
					C - NS: DLPFC, VLPFC, OLPFC, STG, AI				
de Quervain et al., 2004	Punishment, reward	TG	C, UP, N, NS	PET scan	(C + UP) - (N + NS): CdN, thalamus	CdN activation (UP - N) positively correlated with amount invested in punishment (C - N)	Receive unfair offer	Pay to reduce partner's payout or take no action	Personal payout vs. partner's payout
					C - UP: VMPFC, OFC				
White et al., 2014	Fairness, punishment	UG	U, F, C, CA	fMRI	U - baseline: DMPFC	Significant overlap was reported in modulation of activity within DMPFC and AI by offer unfairness and punishment delivered	Reject unfair offer	Pay to reduce partner's payout or take no action	Personal payout vs. partner's payout
					C - baseline: DMPFC, AI, CdN, PAG				
Crockett et al., 2013	Punishment, retaliation, fairness	UG	U, F, NS, V, SP, TP	ATD, fMRI	F - U: VS, MPFC	ATD reduced VS responses to fairness compared to placebo. DS activity on ATD positively correlated with rejection rate	Receive or reject unfair offer	Pay to reduce partner's payout or take no action	Personal payout vs. partner's payout. Punish as second party or third party
					U-ATD - U-placebo: DS				
Knoch et al., 2006	Punishment, Fairness	UG	U, F, NS	Low frequency rTMS	rTMS to right DLPFC: lowered response time, increased rate of acceptance of unfair offers	N/A	Reject unfair offers	Reject or accept offers	Personal payout vs. partner's payout
**References^a^**	**Phenomenon**	**Task**	**Conditions^b^**	**Technique**	**Main results**	**BOLD signal predictability**	**Inequity aversion**	**Cost-benefit calculation**	**Social reference frame**
Koenigs and Tranel, 2007	Fairness, emotion	UG	U, F	Bilateral VMPFC lesion patients	VMPFC group accepted fewer unfair offers	N/A	Reject unfair offers	Reject or accept offers	Personal payout vs. partner's payout
Ruff et al., 2013	Punishment, social norm compliance	DG	FP: N, UP, NS	Anodal, cathodal, or sham tDCS to the right DLPFC	Anodal tDCS (increased excitability) increased magnitude of unfair offers made; cathodal tDCS (reduced excitability) decreased magnitude of unfair offers made	N/A	Make unfair offer	Threat of punishment from opponent	Punishment expected from opponent

*ACC, anterior cingulate cortex; AI, anterior insula; AMYG, amygdala; ATD, acute tryptophan depletion; C, costly punishment; CA, costly acceptance; CdN, caudate nucleus; DG, dictator game; DLPFC, dorsal lateral prefrontal cortex; DS, dorsal striatum; F, fair offer; fMRI, functional magnetic resonance imaging; FP, first person; GSR, galvanic skin response; HR, heart rate; HRV, heart rate variation; N, no punishment; NAc, nucleus accumbens; NS, nonsocial (computer); OFC, orbitofrontal cortex; PAG, periaqueductal gray; PET, positron emission tomography; rTMS, repetitive transmagnetic stimulation; SP, second party; STG, superior temporal gyrus; tDCS, transcranial direct cranial stimulation; TG, trust game; TP, third party; U, unfair offer; UG, ultimatum game; UP, uncostly punishment; V, view offers; VLPFC, ventrolateral prefrontal cortex; VMPFC, ventromedial prefrontal cortex; VS, ventral striatum*.

a *In the case of a publication with multiple experiments, the most relevant experiment to the review is included in the table*.

b *Conditions are generalized to best reflect the situation of the condition, even if it is named differently in the study. Participants are the second party (receiving an offer) in a social situation unless otherwise noted*.

Furthermore, converging evidence suggests that prefrontal regions are specialized for evaluating fairness and guiding norm compliance during punishment. In addition to the previously cited work implicating the DLFPC in administering punishment as the SP, low-frequency repetitive transmagnetic stimulation to the right DLPFC increased the acceptance rate of unfair offers in the UG (Knoch et al., 2006). By contrast, participants with bilateral lesions in the ventromedial prefrontal cortex (VMPFC) exhibited the opposite pattern, accepting fewer unfair offers (Koenigs and Tranel, 2007). Furthermore, increasing or decreasing neuronal excitability using transcranial direct current stimulation in the right lateral prefrontal cortex (rLPFC) differentially controls sanction-free compared to sanction-induced transfers, without affecting the conceptualization of fairness norm and sanctions, when a player makes a monetary offer in either a dictator or UG context (Ruff et al., 2013). These results demonstrate that rLPFC is causally involved in norm compliance, but in strikingly different ways depending on the presence of punishment threats.

However, it remains unclear what computations are primarily driving the neural signals in altruistic punishment tasks. In the following sections, we highlight three fundamental cognitive processes occurring in most altruistic punishment scenarios that might play significant roles in driving the neural activations. These processes may inform how different neural circuits are implemented in driving altruistic punishment.

## Inequity aversion

**Figure 1:**
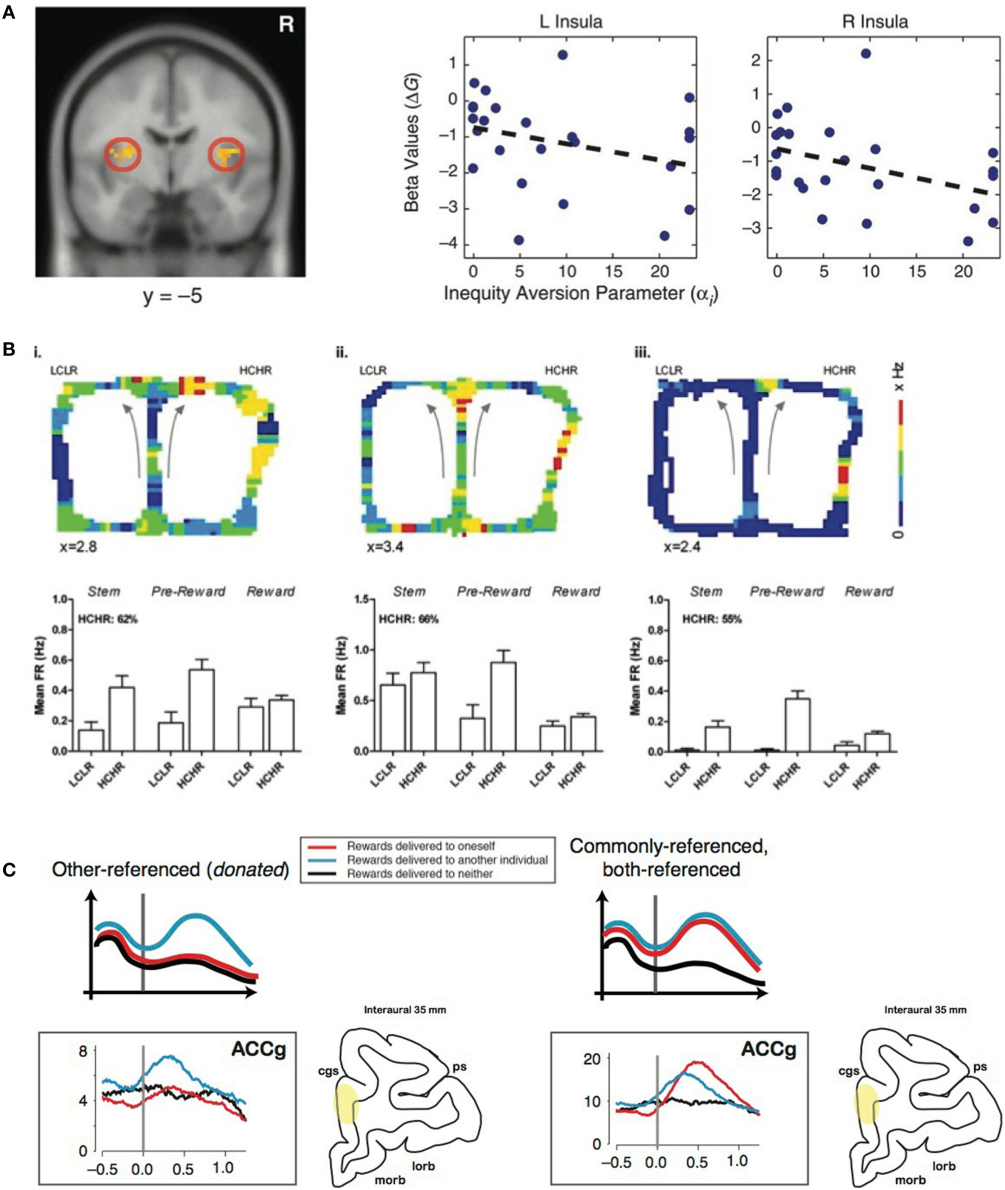
**Representative studies on the neural components of altruistic punishment. (A)** Left: Hsu et al. (2008) found that activation in the bilateral insula (left) is negatively correlated with inequity in a task involving allocating differential benefits across two groups of children. Right: Individual's activations (beta values) were negatively correlated with individual inequity-aversion parameters. Modified from Hsu et al. (2008) with permission. **(B)** In the study by Hillman and Bilkey (2010), mice navigated through a maze in which they either chose a “low cost, low reward” (LCLR) arm (left) or a “high cost, high reward” (HCHR) arm (right). For each of the three typical HCHR-biased ACC cells shown here (i–iii), firing rates that are pseudo-color mapped onto the spatial position of the maze illustrate overall higher activity for the HCHR compare to the LCLR option. The bar graphs show the mean firing rates (FR) across different spatial positions (“epochs”) for HCHR and LCLR choices for each cell. The percentage of HCHR choices is also indicated. Modified from Hillman and Bilkey (2010) with permission. **(C)** Left: Other-referenced representation of rewards allocated to another monkey in the room found in the subpopulation of neurons in the gyrus of the anterior cingulate cortex (ACCg). Right: Mirrored (commonly-referenced) representation of rewards received by an actor and another monkey found in the subpopulation of ACCg neurons. Modified from Chang et al. (2013) and Chang (2013), with permission.

Altruistic punishment occurs in response to an offer perceived to be inequitable. Inequity often evokes negative affect, which may motivate the decision to altruistically punish (Montague and Lohrenz, 2007). Using a resource-sharing task, Haruno and Frith (2010) showed that BOLD signals from amygdala (AMYG) predict individual differences in aversion to inequity, such that prosocial orientation is driven by an intuitive aversion for the inequitable division of resources across self and others. Furthermore, in a task asking participants to allocate goods between two groups of children, high insula activity was associated with choosing an equitable allocation vs. an inequitable one, supporting the role of the insula for signaling inequity associated with a norm violation (Hsu et al., 2008) (Figure 1A). This finding suggests that individuals with stronger negative affective signaling from the bilateral AI reduce inequity due to a greater sensitivity to the violation of fairness (Hsu et al., 2008). Unreciprocated cooperation, which could be conceptualized as a violation of one's expectation based on the social norm, also increases activation in the bilateral AI, as well as the left AMYG (Rilling et al., 2008). AMYG activity during a decision-making task with losses and gains correlated with loss aversion (Sokol-Hessner et al., 2013). Taken together, social exchanges with an inequitable outcome seem to recruit neural systems, including the AI and AMYG, involved in affective signaling to influence norm compliance by modulating fairness perception.

Because inequity motivates a punishing action, neural processes associated with inequity detection and aversion could significantly guide the decision to costly punish. The aversion associated with inequity, on the other hand, may reflect how the neural signals corresponding to inequity detection are transformed and further processed by emotion-related circuitry. Converging evidence indeed indicates that task events correlated with inequity typically activate brain regions implicated in affective processing. Further research is needed to understand how these signals influence the decision to punish.

In realistic settings, there are not necessarily tight temporal relations between the time of inequity detection and the punishment—some punishments may occur as an immediate reaction, whereas others may occur long after the infraction, many days and even years following inequity detection. In the laboratory, most studies have had focused on relatively small time window between inequity detection and punishment for practical purposes. The role of inequity-evoked negative affect is likely to be greater when the time from the inequity detection to the punishing act is relatively short. It would be interesting to test the magnitude of affective drive in different brain regions during punishing decisions as a function of the delay between the time of inequity detection and forming the decision to punish.

## Cost-benefit calculation

Cost-benefit analysis could occur when deciding to carry out altruistic punishment, requiring that the cost of punishment be weighed against the benefit of punishing (Egas and Riedl, 2008). The costly punishment decision could hinge upon the value representation of the possible outcomes (Sugrue et al., 2005), which subsequently may depend on the severity of the opponent's infraction, the ratio of monetary cost to punish, the impact of the punishment itself on the opponent, as well as any expected future gains from punishing.

In examining effort as a proxy for cost required for some benefit, a lesion to ACC reduced a rat's willingness to expend effort to receive a large reward, whereas control animals typically expended energy (Walton et al., 2003). When human subjects performed a similar effort-based task, BOLD activity in the dorsal ACC and striatum increased with respect to the net value of the outcome rather than the amount of effort anticipated (Croxson et al., 2009). Single-unit recordings in rat ACC during a choice task of two options differing in cost-benefit ratio found a population of neurons with elevated firing rates during the pursuit of a high cost, high reward option, an effect that did not solely reflect variations in physical effort or food acquisition, suggesting the ACC's role in integrating information about cost and reward (Hillman and Bilkey, 2010) (Figure 1B). Furthermore, in a competitive setting, a population of ACC neurons signals the choice with the greatest net utility based on the cost of competing and the benefit of reward (Hillman and Bilkey, 2012). Inactivating ACC in mice decreases their preference for the option requiring greater effort (Hosking et al., 2014). Although such efforts related to acquiring a reward or completing a task may not be directly translatable to the monetary loss in costly punishment, they share the basic principle of incurring a cost to potentially obtain a desired outcome.

Some studies have related a nonsocial reward currency with social rewards, which could be occurring in altruistic punishment when a player sacrifices money to punish another player. An exchange rate between viewing social images and receiving monetary rewards that falls along a distribution correlated with the valuation of the image is reflected in the hemodynamic activity of the posterior VMPFC (Smith et al., 2010). Functional connectivity of the VMPFC to temporal-parietal junction, MPFC, middle temporal gyrus, and posterior cingulate cortex suggests a network governing subjective valuations of social reward (Smith et al., 2014). Weighing the cost and benefit in the social reward domain is not a behavior restricted to humans. Rhesus macaques also are willing to exchange small amounts of juice rewards in order to view socially salient images but require juice overpayment to view less desirable images (Deaner et al., 2005). Importantly, the specific amount of juice sacrificed or demanded falls along a distribution that is correlated with the valuation of the image, generating an exchange rate between social (image) and nonsocial (juice) rewards. Similar to such an exchange rate between monetary (or primary reinforcer in monkeys) and social reward, participants forgo a monetary reward to punish in SP or TP punishment, in which the loss is compensated by the reduction in their opponent's endowment. If punishing the defector is in fact rewarding (de Quervain et al., 2004), the punishing cost could be thought in part as being exchanged with a social reward.

Regardless of whether the gains of altruistic punishment are monetary or social, cost-benefit calculations may play a key role in the neural processes leading up to a decision to punish at a predicted cost. Examining cost-benefit relations in both nonsocial rewards and social rewards and associated neural activity across different individuals during costly punishment decisions may reveal intriguing insights into the mechanisms underlying the decision to costly punish.

## Social reference frame

Any behavior that involves another individual requires a set of representations across self and others. Costly punishment, in particular, may require the evaluation of others' internal states when an individual is faced with outcomes that could ultimately benefit another individual or a group. Evidence demonstrates that there are shared networks in the brain that compute information with respect to self and others (Decety and Sommerville, 2003). Importantly, these shared representations seem to be generalized to multiple cognitive domains. The brain regions related to the affective response to pain, including the rostral portion of ACC, bilateral insula (both medial and anterior), and the brainstem, are activated in groups who either receive an electric shock to their own finger or watch a significant other receive a shock (Singer et al., 2004). Furthermore, it was shown that observing another person experiencing pain activates a broad range of brain areas across frontal and parietal cortices as well as AMYG, areas that are implicated in emotional and social cue processing (Ochsner et al., 2008). Likewise, a TP observer could take on a SP perspective such that another player's misfortune is construed as his own. A recent study suggests that some brain areas may process self-specific misfortune but other areas may project other's misfortune onto one's own. Corradi-Dell'Acqua et al. (2013) found selective activation in MPFC when participants were shown unfair offers involving themselves but not others, whereas AI activations were also associated with unfair offers to others (2013). Whether “perspective-taking” or “affective projection” is a prerequisite to promoting TP remains to be explored.

In addition, the TP observer likely infers the reward contingencies from the perspectives of the two players after punishing. Such multidimensional inferences across oneself and another individual must require a signal transformation across self and others (Chang, 2013) (Figure 1C). Insight into this transformation could be obtained by examining how reward outcomes across self and others are encoded in the brain. A recent study tested such encoding mechanisms when pairs of rhesus macaques were engaged in a social reward exchange paradigm (Chang et al., 2013). When an actor monkey was choosing to deliver juice rewards between himself and a recipient (Self/Other) as well as between the recipient and no one (Other/Neither), anatomically distinct regions of the primate frontal cortex encoded the reward outcomes across self and others using different social reference frames. Neurons in OFC primarily signaled the received rewards of the actor monkey, whereas neurons in the sulcus of ACC (ACCs) predominantly signaled the foregone rewards of the actor. Notably, one subpopulation of neurons in ACC gyrus (ACCg) exclusively signaled the rewards received by the recipient, whereas another subpopulation mirrored the rewards received by the actor or the recipient. In addition to these “other-regarding” neurons, another subpopulation in ACCg exclusively encoded one's own reward outcomes. The specialized function of ACCg in signaling the rewarding events of others was also recently reported in a human neuroimaging study (Apps and Ramnani, 2014). An accurate readout of self- and other-referenced reward information could be critical for mediating the concept of actor and recipient during altruistic punishment.

In both SP and TP punishment, the punisher must be able to accurately process affective responses and reward outcomes as a result of punishment across himself and the other player or players involved. As such information is essential to behaviors directed at other individuals, it is critical to comprehend the amount and specific nature of neural activations driven by such computations in social punishment scenarios.

## Concluding remarks

Costly punishment poses some of the most interesting evolutionary questions for scientists. Cooperation, which rests inherently on the shared needs of individuals and groups, seems to be at complete odds with traditional evolutionary theories that pit individuals against each other in a race for survival and reproduction. Yet cooperation in animal and human behavior often determines the survival of entire populations. The neural underpinnings of altruistic punishment in humans are actively being investigated, but many questions remain unanswered. Several studies so far suggest the lateral aspect of the prefrontal cortex as a key locus in mediating altruistic punishment (Knoch et al., 2006; Spitzer et al., 2007; Strobel et al., 2011; Ruff et al., 2013). These regions may reflect the computations of cognitive variables. However, some studies also highlight the importance of the insula and AMYG for their role in affective processing during such punishments (Hsu et al., 2008; Haruno and Frith, 2010). The neural processes related to these cognitive and affective variables are likely to interact with one another up until the time of punishment. Such ongoing interactions may provide the basis for internal deliberation on a decision to punish, as well as mediating a potential change of mind before the execution of that decision.

A paradigm with high temporal sensitivity may reveal how cognitive and affective signals in the brain converge or diverge across the entire timespan of deciding to punish at a cost. For example, future investigations combining fMRI with another method allowing for a higher temporal resolution, such as electroencephalography or functional near-infrared spectroscopy, may reveal new, crucial information on the region-to-region interactions between neural signals correlated with inequity aversion, cost-benefit calculation, and information processing across self and others. Furthermore, developing a nonhuman primate model of costly punishment may complement research in humans by providing more detailed neuronal mechanisms of the three core neural processes involved in altruistic punishment through single-unit recording and pharmacological interventions of specific populations of neurons. Another important factor to consider in any social neuroscience research is the context in which a given social behavior takes place. Understanding how social context gates the neural processes associated with inequity aversion, cost-benefit calculation, and information processing across self and others will better inform the complex contingencies behind altruistic punishment.

### Conflict of interest statement

The authors declare that the research was conducted in the absence of any commercial or financial relationships that could be construed as a potential conflict of interest.
